# Influence of lifestyle choices on risks of CYP1B1 polymorphisms for prostate cancer

**DOI:** 10.1111/jcmm.13696

**Published:** 2018-08-22

**Authors:** Taku Kato, Yutaka Hashimoto, Ryan K. Wong, Yozo Mitsui, Shigekatsu Maekawa, Inik Chang, Varahram Shahryari, Soichiro Yamamura, Shahana Majid, Sharanjot Saini, Z. Laura Tabatabai, Rajvir Dahiya, Takashi Deguchi, Yuichiro Tanaka

**Affiliations:** ^1^ Department of Urology Veterans Affairs Medical Center San Francisco CA USA; ^2^ Department of Urology University of California San Francisco CA USA; ^3^ Department of Urology Gifu University Graduate school of Medicine Gifu Japan; ^4^ Department of Oral Biology Yonsei University College of Dentistry Seoul South Korea; ^5^ Department of Pathology Veterans Affairs Medical Center and University of California San Francisco CA USA

**Keywords:** alcohol consumption, cytochrome P450 1B1, polymorphisms, prostate cancer, smoker

## Abstract

Cytochrome P450 1B1 (CYP1B1) converts xenobiotics to carcinogens and how lifestyle choices may interact with CYP1B1 polymorphisms and affect prostate cancer risk was assessed. Blood genomic DNA from a Caucasian population was analysed at polymorphic sites of the 5′ untranslated region of CYP1B1 using TaqMan genotyping assays. Overall, drinker status and minor alleles at rs2551188, rs2567206 and rs10175368 were associated with prostate cancer. Linkage was observed between rs2551188, rs2567206, rs2567207 and rs10175368, and the G‐C‐T‐G haplotype (major allele at respective sites) was decreased in cancer. Interestingly when classified by lifestyle factors, no associations of genotypes were found for non‐smokers and non‐drinkers, whereas on the contrary, minor type at rs2567206 and rs10175368 increased and major G‐C‐T‐G decreased risk for cancer among smokers and drinkers. Interestingly, rs2551188, rs2567206 and rs10175368 minor genotypes correlated with increased tissue CYP1B1 as determined by immunohistochemistry. Further, rs10175368 enhanced luciferase activity and mobility shift show stronger binding of nuclear factor for the minor allele. These results demonstrate smoking and alcohol consumption to modify the risks of CYP1B1 polymorphisms for prostate cancer which may be through rs10175368, and this is of importance in understanding their role in the pathogenesis and as a biomarker for this disease.

## 1 INTRODUCTION

Worldwide, prostate cancer ranks second in incidence rates and fifth in deaths from cancer among men.^1^ In the United States however, rates are dramatically high, ranking first in incidence with an estimated 164 690 new cases and second in deaths with 29 430 expected.^2^ The probability of developing invasive cancer increases with age from 0.2% in men 49 years or less, 1.7% (50‐59 years), 4.8% (60‐69 years) and 8.2% (≥70 years). Despite being a disease of high morbidity and mortality, the cause is not well understood and identifying risks in the carcinogenesis process is an important step towards its prevention.

Lifestyle factors such as tobacco smoking and alcohol consumption are established risks for various types of cancers.^3^, ^4^ Worldwide, tobacco smoking accounts for roughly 21% of cancer deaths with 29% in high‐income countries.^3^ In the USA in the year 2010, the estimated death rate of all cancers due to cigarette smoking was roughly 38% with about 112 000 deaths among men aged 35 years or older and does not include additional deaths from environmental tobacco smoke or usage of cigars, pipes or smokeless tobacco.^5^ In prostate, a meta‐analysis of 4 million cohort participants showed that current cigarette smoking was correlated with increased risk of cancer death (relative risk [RR]; 1.24, 95% confidence interval [CI]; 1.18–1.31) with cigarettes smoked per day having a dose‐response association with cancer mortality.^6^ Also, compared with non‐smokers, former smokers (hazard ratio [HR]; 1.63, 95% CI; 1.30–2.04, *P* < .001) and current smokers (HR; 1.80, 95% CI 1.45–2.24, *P* < .001) had a higher risk of prostate cancer biochemical recurrence.^7^

Alcohol consumption accounts for about 5% of all cancer deaths worldwide and a large proportion of cancers in Europe and America.^3^ In the USA, 92% of respondents 18 years and older claimed life‐time alcohol usage^8^ and up to 3.7% of cancer deaths were linked to drinking in the USA.^9^ In a meta‐analyses study, Bagnardi et al^4^ find alcohol drinking to be associated with various cancers and this effect is strongest among heavy drinkers. The effect of alcohol drinking appears to be dose‐dependent as light to moderate drinking resulted in a lower risk, whereas heavy drinking caused an increased risk of certain cancers.^10^ As for prostate, a dose‐response was also observed for cancer risk among current drinkers (*P*_trend_ **< .01).^11^

Tobacco smoking and alcohol thus play a causative role in the carcinogenesis process, and a major enzyme that affects this process is cytochrome P450 (CYP) 1B1. CYP1B1 is a member of the CYP superfamily involved in phase I metabolism of many xenobiotics.^12^, ^13^ CYP1B1 can metabolically convert tobacco smoke pro‐carcinogens such as polycyclic aromatic hydrocarbons to reactive or carcinogenic intermediates^13^, ^14^ and result in DNA adduct formation.^14^, ^15^ In prostate, mRNA transcripts including CYP1B1 were observed along with DNA adducts after incubating with 2‐amino‐1‐methyl‐6‐phenylimiazo[4,5‐b]pyridine (PhIP), 2‐amino‐3‐methylimidazo[4,5‐f]quinoline (IQ) and benzo[a]pyrene (B[a]P).^14^ Also in primary mammary epithelial cells, B[a]P caused DNA adducts to form as well as CYP1B1 induction.^15^ Alternatively, PAHs or smoking can enhance levels of CYP1B1 expression.^13^, ^16^ As CYP1B1 is expressed in human prostate,^17^ gene up‐regulation and activation of pro‐carcinogens may thus be influential in the prostatic carcinogenesis process.

The main form of alcohol in alcoholic beverages is ethanol, which may pose a risk even at moderate drinking amounts.^18^ Levels of ethanol in blood were shown to dramatically rise above 15 mmol/L within 30 minutes after drinking whisky (0.72 g/kg ethanol) and gradually decreased over a 6 hour period.^19^ Although the tumorigenicity of ethanol itself may be dependent on experimental conditions, its direct metabolic product acetaldehyde has been shown to be carcinogenic in animals.^20^ Indeed, CYP1B1 was shown to metabolize ethanol into significant amounts of acetaldehyde^21^ and studies in rats show inhalation and oral administration of acetaldehyde to be carcinogenic in animals.^22^, ^23^ Acetaldehyde can interfere with DNA synthesis and repair,^24^ cause point mutations,^20^, ^24^ form direct bonds with DNA^24^ and form other DNA adducts at cellular concentrations.^24^, ^25^ The International Agency for Research on Cancer (IARC) has listed acetaldehyde as carcinogenic.^18^ Additionally, acetaldehyde gets metabolized into acetate via acetaldehyde dehydrogenase and in the process, radicals are formed^24^ that can bind to DNA.^26^

It is thus apparent that CYP1B1 can lead to cancer by activating various compounds into carcinogenic forms and of interest are genetic polymorphisms that can alter enzyme levels. A number of studies have focused on the coding region or missense variants that enhance CYP1B1 catalytic activity and show them to be associated with prostate cancer.^27^, ^28^ Also of importance are variants in the promoter or 5′ untranslated region (5′UTR) as these may lead to an up‐regulation^29^ or down‐regulation^30^ of RNA transcription and consequentially, enzyme expression levels. A study on promoter polymorphisms and prostate cancer did indeed show variants to be associated with progression of cancer.^31^ Promoter polymorphisms may thus be indicators of disease susceptibility or factors in polygenic diseases due to alterations in enzyme expression.

To date, studies on polymorphic variants of the CYP1B1 promoter region/5′UTR and their risks for prostate cancer and their functional role are lacking. Additionally, the impact of lifestyle factors on the risks of these variants for prostate cancer has never been investigated. In this report, we evaluated the risks of 8 polymorphic sites in the promoter region/5′UTR of CYP1B1 for prostate cancer and how this is influenced by major lifestyle factors among a Caucasian population. We have been suggested that minor genotypes and alleles are associated with cancer and that tobacco smoking or alcohol consumption can increase their risk. Also, we examined the functional effects of polymorphisms and have been suggested that minor alleles can affect promoter activity as well as correlate with protein expression in prostatic cells.

## 2 MATERIAL AND METHODS

### 2.1 Subjects and specimens

The population study consisted of Caucasian men from the Northeast region of the USA. From these subjects, blood DNA was procured by the biorepository of Bioserve (Beltsville, MD). All men gave written informed consent for research purposes. The signed forms and patient records are kept at the collecting medical institution, with BioServe never receiving or maintaining any personal health information with the identity of the donor. Donor samples were completely anonymized. At the laboratory in San Francisco, specimens and de‐identified patient data were obtained from Bioserve and used for analyses. Characteristics of subjects included in the case‐control study are summarized in Table 1. A total of 400 samples were obtained from patients with sporadic prostate cancer. Stage of prostate cancer was according to the American Joint Committee on Cancer (AJCC) classification (https://www.ncbi.nlm.nih.gov/pubmedhealth/PMH0032726/). To assess risks for prostate cancer, specimens were also obtained from 405 men identified as healthy. From all specimens, additional information regarding lifestyle choices was obtained: Smoker is identified as those who currently smoke or are former smokers, and drinker is those who recognize themselves as drinker of alcohol. All patients with prostate cancer and healthy volunteers were thus of the same race (Caucasian), sex (male), age‐matched and from the same region within the USA.

**Table 1:** Patient characteristics of case‐control study. Values expressed as mean ± SD

	Control (n = 405)	Prostate cancer (n = 400)	*P*‐value	Method
Age (y)	68.3 ± 4.9	68.9 ± 3.8	.188	Student *t* test
Range (y)	59‐80	44‐91		
BMI	26.7 ± 8.1	27.1 ± 4.1	.107	Mann‐Whitney U test
Smoking status				
Non‐smoker	161 (39.8%)	137 (34.3%)	.109	Chi‐square
Current or former smoker	244 (60.2%)	263 (65.8%)		
Alcohol consumption				
Non‐drinker	217 (53.6%)	175 (43.8%)	**.006**	Chi‐square
Drinker	185 (45.7%)	222 (55.5%)		
Unknown	3 (0.7%)	3 (0.7%)		
Stage				
I		97 (24.3%)		
II		156 (39.0%)		
III		53 (13.3%)		
IV		12 (3.0%)		
Unknown		82 (20.5%)		

*P* < .05 are in bold.

In addition, for CYP1B1 expression analyses, 83 samples of benign prostatic hyperplasia (BPH) were collected from the Department of Anatomy and Pathology at the Veterans Affairs Medical Center in San Francisco. Specimens are formalin‐fixed paraffin‐embedded and de‐identified data attached to patients were obtained. Patients include 51 Caucasians, 14 African‐Americans, 10 Asians, 1 Hispanic, 1 native Hawaiian and 6 of unknown race. Average age ± SD of these patients was 68.18 ± 8.13 years (range 41 to 87).

This study was approved by the Clinical Research Office of the San Francisco Veterans Affairs Medical Center and the Institutional Review Board of the University of California at San Francisco.

### 2.2 CYP1B1 genotyping

To analyse CYP1B1 polymorphisms, TaqMan genotyping assays (Applied Biosystems, Foster City, CA) were utilized according to manufacturer's instructions. In brief, a 5‐μL reaction containing TaqMan Universal Master Mix (Applied Biosystems) and 0.5 ng of sample DNA was prepared. Thermal cycle conditions were 95°C for 10 minutes, followed by 40 to 60 cycles of 95°C for 15 seconds and 60°C for 1 minute. End‐point fluorescent readings were analysed using the QuantStudio 7 Real‐Time PCR system (Applied Biosystems). The polymorphic sites analysed were in the promoter region/5′UTR of the CYP1B1 gene which were reported in dbSNP (http://www.ncbi.nlm.nih.gov/projects/SNP/) and ID# (base change, distance prior to ATG start site) are as follows: rs2551188 (G to A, −263 bp), rs2567206 (C to T, −1001 bp), rs2567207 (T to C, −1112 bp), rs162556 (T to C, −3924 bp), rs10175368 (G to A, −5331 bp), rs163090 (T to A, −11102 bp), rs162330 (T to G, −16966 bp) and rs162331 (A to G, −17064 bp).

### 2.3 Cell culture

Caucasian prostate cancer PC3 and DU145 cell lines were obtained from American Type Cell Collection (ATCC, Manassas, VA, USA). Cells were cultured in RPMI1640 medium supplemented with 10% foetal bovine serum in a humidified atmosphere containing 5% CO2. These cell lines were authenticated by DNA short‐tandem repeat analysis by ATCC, and experiments with cell lines were performed within 6 months of their revival.

### 2.4 Immunohistochemical analysis

Immunostaining of CYP1B1 was performed on specimens of BPH. Slides consisting of 4 μm slices of tissue underwent the protocol of the UltraVision Detection System (Thermo Fisher Scientific, Waltham, MA) according to manufacturer's instructions. After 12‐hour incubation with rabbit monoclonal antibody for CYP1B1 (1:500 dilution, #ab185954, Abcam, Cambridge, UK), 3, 3′‐diaminobenzidine (DAB) was added as chromogen followed by counterstaining with haematoxylin. Cellular expression levels were analysed by the intensity of positive cells using ImageJ software (http://rsb.info.nih.gov/ij) and ranked on an overall scale from 0 to 3, with 0 indicating the absence of staining; 1, weak staining; 2, moderate staining; and 3, strong staining.

### 2.5 Site‐directed mutagenesis and promoter reporter assay

Two sets of *Gaussia* luciferase CYP1B1 vectors consisting of the 5′UTR or promoter region at the following base pairs prior to the ATG start site: −1143 to +190 and customized −5590 to −5090 were utilized along with negative control (Gene Copoeia, Rockville, MD). These underwent site‐directed mutagenesis using QuikChange ‖ XL Site‐Directed Mutagenesis Kit (Agilent technologies, Santa Clara, CA) according to manufacturer's protocol. For the −1143 to +190 region vector, primer sequences for mutagenesis included rs2551188 sense, 5′‐GGTGTCCCCAGAACTACGCTCGGTACAAC‐3′ and antisense, 5′‐GTTGTACCGAGCGTAGTTCTGGGGACACC‐3′; and rs2567206 sense, 5′‐CACCCTCGGCTGTGCACGCACAGTC‐3′ and antisense, 5′‐GACTGTGCGTGCACAGCCGAGGGTG‐3′. In vector containing the −5590 to −5090 region, mutagenesis primers were rs10175368 sense, 5′‐GATGTATCTTAGAGTCAATGATGCAATTATAATTGGTAGCTTCCTTT‐3′ and antisense, 5′‐AAAGGAAGCTACCAATTATAATTGCATCATTGACTCTAAGATACATC‐3′. Mutagenesis was amplified by pfu HF DNA polymerase using thermal cycling conditions of 95°C for 1 minute, followed by 18 cycles of 95°C (1 minute), 55°C (1 minute) and 68°C (20 minutes), then 68°C for 7 minutes. Amplified product was exposed to Dpn I at 37°C for 1 hour, and the nicked mutant plasmid DNA was transformed into XL10‐Gold Ultracompetent cells for repair. Plasmids were then checked for accuracy by DNA sequencing (TACGen, San Pablo, CA). The FASTA sequences and polymorphism information were obtained from the NCBI.

DU145 and PC3 cells in 96‐well plates were transfected with 100 ng of reporter gene DNA constructs using Lipofectamine 2000 reagent (Invitrogen). After 72 hours, secreted *Gauccia* and alkaline phosphatase (ALP) luciferase activities were determined using the Detects *Gauccia* luciferase and secreted Alkaline phosphatase kit (Gene Copoeia) according to manufacturer's instructions and Victor™ X2 luminometer (Perkin Elmer, Waltham, MA). Activity levels of *Gauccia* were normalized to ALP.

### 2.6 Detection of DNA‐protein complex

Nuclear extract was collected from DU145 cells using NE‐PER Nuclear and Cytoplasmic Extraction Reagents kit (Thermo Fisher Scientific) according to manufacturer's protocol. To determine the binding capability of the nuclear component, extracts (5 μg) along with biotin‐labelled (40 fmol) with and without unlabelled (4 pmol) oligonucleotide probe were incubated at room temperature for 20 minutes. Oligonucleotide probe sequences were as follows: rs10175368 (major allele sense, 5′‐TTATAATTGCATCATCGACTCTAAGATACA‐3′ and anti‐sense, 5′‐AATATTAACGTAGTAGCTGAGATTCTATGT‐3′; minor allele sense, 5′‐TTATAATTGCATCATTGACTCTAAGATACA‐3′ and anti‐sense, 5′‐AATATTAACGTAGTAACTGAGATTCTATGT‐3′). Probes were annealed in TEN buffer by gradually decreasing temperature from 95 to 25°C using thermal cycler prior to adding to reaction mixture. DNA‐protein complexes were separated on 6% DNA retardation gel (Invitrogen), blotted onto Biodyne B Pre‐Cut modified nylon membranes (0.45 μm, Thermo Fisher Scientific) and visualized by ChemiDoc XRS+ system (Bio‐Rad, Hercules, CA).

### 2.7 Statistical analyses

For genotypic differences, multiple logistic regression analyses adjusting for age, body mass index (BMI) and smoker and/or drinker status were calculated for adjusted odds ratio (OR) and 95% confidence intervals (CI) along with Wald's test, using R package, epiDisplay (https://CRAN.R-project.org/package=epiDisplay). Allelic distributions were analysed by Fisher's exact test and OR (95% CI) calculated using the IBM SPSS Statistics version 23 software (IBM, Armonk, NY). Linkage disequilibrium between locations was measured in healthy control samples, and haplotype frequency differences were calculated using SNPAlyze version 6.6.1 software (DYNACOM; Tokyo, Japan). Hardy‐Weinberg equilibrium was also determined for each site among healthy controls.

Differences in protein expression levels were analysed by two‐tailed Mann‐Whitney U test, whereas for luciferase reporter, two‐tailed Student *t* and Dunnett *t* tests were utilized. Also, differences in patient characteristics were analysed by the following: age—two‐tailed Student *t* test, BMI—two‐tailed Mann‐Whitney U test, smoker status—chi‐square test and alcohol drinker status—chi‐square test.

Analyses were carried out at least in triplicate, and *P* < .05 was considered statistically significant.

## 3 RESULTS

### 3.1 Lifestyle choices and prostate cancer risk

Risks of prostate cancer for patient characteristics such as age, BMI, smoker status and alcohol drinker status are shown in Table 1. Alcohol consumption was observed to be a risk for cancer with 55.5% of drinkers developing cancer compared to 45.7% without this disease (*P* = .006). No associations were observed for age, BMI or smoker status.

### 3.2 CYP1B1 polymorphisms and prostate cancer risk

Table 2 shows the genotypic and allelic frequencies for the 8 polymorphic sites of the CYP1B1 gene analysed in prostate cancer and healthy controls. Genotypic frequencies at all sites with exception of rs2567207 are in agreement with the Hardy‐Weinberg equilibrium (data not shown). Interestingly in this Caucasian population, the minor genotype at rs2567206 (*P* = .025) and rs10175368 (*P* = .004) for a dominant pattern was observed to be a significant risk for prostate cancer. The adjusted OR for C/T+T/T compared with major genotype C/C was 1.38 with a 95% CI of 1.04 to 1.83 for rs2567206 and at rs10175368, G/A+A/A compared with major G/G had an adjusted OR of 1.52 with 95% CI of 1.14 to 2.02. Likewise, the minor allele T and A were also observed to be significantly increased in PC at rs2567206 (*P* = .008) and rs10175368 (*P* = .001), respectively. Additionally, minor allele A at rs2551188 proved to be a risk for cancer (*P* = .043). No differences were found between patients with prostate cancer and control at other polymorphic sites.

**Table 2:** Genotypic (dominant pattern) and allelic frequencies of CYP1B1 polymorphisms in healthy control and prostate cancer patients

SNP ID	Genotype	Control n (%)	Cancer n (%)	Adj OR^a^ (95% CI)	Wald's test	Allele	Control n (%)	Cancer n (%)	OR (95% CI)	Fisher's exact
rs2551188	G/G	226 (55.8)	201 (50.2)	Reference 1.23 (0.93‐1.63)	0.153	G	612 (75.6)	568 (71.0)	Reference 1.26 (1.01‐1.58)	**0.043**
	G/A+A/A	179 (44.2)	199 (49.8)			A	198 (24.4)	232 (29.0)		
rs2567206	C/C	230 (56.8)	192 (48.0)	Reference 1.38 (1.04‐1.83)	**0.025**	C	615 (75.9)	560 (70.0)	Reference 1.35 (1.08‐1.69)	**0.008**
	C/T+T/T	175 (43.2)	208 (52.0)			T	195 (24.1)	240 (30.0)		
rs2567207	T/T	214 (52.8)	203 (50.7)	Reference 1.09 (0.82‐1.44)	0.567	T	601 (74.2)	566 (70.8)	Reference 1.19 (0.96‐1.48)	0.132
	T/C+C/C	191 (47.2)	197 (49.3)			C	209 (25.8)	234 (29.2)		
rs162556	T/T	98 (24.2)	109 (27.3)	Reference 0.84 (0.61‐1.16)	0.296	T	402 (49.6)	413 (51.6)	Reference 0.92 (0.76‐1.12)	0.426
	T/C+C/C	307 (75.8)	291 (72.8)			C	408 (50.4)	387 (48.4)		
rs10175368	G/G	242 (59.8)	195 (48.8)	Reference 1.52 (1.14‐2.02)	**0.004**	G	630 (77.8)	561 (70.1)	Reference 1.49 (1.19‐1.87)	**0.001**
	G/A+A/A	163 (40.2)	205 (51.2)			A	180 (22.2)	239 (29.9)		
rs163090	T/T	100 (24.7)	106 (26.5)	Reference 0.91 (0.66‐1.25)	0.546	T	415 (51.2)	389 (48.6)	Reference 1.11 (0.91‐1.35)	0.296
	T/A+A/A	305 (75.3)	294 (73.5)			A	395 (48.8)	411 (51.4)		
rs162330	T/T	101 (24.9)	100 (25.0)	Reference 0.99 (0.72‐1.37)	0.965	T	410 (50.6)	389 (48.6)	Reference 1.08 (0.89‐1.32)	0.426
	T/G+G/G	304 (75.1)	300 (75.0)			G	400 (49.4)	411 (51.4)		
rs162331	A/A	92 (22.7)	113 (28.2)	Reference 0.77 (0.56‐1.07)	0.115	A	403 (49.8)	411 (51.4)	Reference 0.94 (0.77‐1.14)	0.517
	A/G+G/G	313 (77.3)	287 (71.8)			G	407 (50.2)	389 (48.6)		

a OR adjusted for age, BMI, smoker and drinker status. *P* < .05 are in bold.

### 3.3 Linkage disequilibrium of CYP1B1 polymorphisms

Linkage between the polymorphic sites of CYP1B1 was calculated and Table 3 shows R^2^‐values among healthy controls. Two sets of polymorphic sites were observed to be in linkage disequilibrium. One group consisted of 4 sites (rs2551188, rs2567206, rs2567207 and rs10175368) and the other with 3 (rs163090, rs162330 and rs162331). No linkages were observed for rs162556.

**Table 3:** Linkage disequilibrium among 8 polymorphisms of CYP1B1 in normal healthy individuals. *R* ^2^‐values shown

SNP ID	rs2551188	rs2567206	rs2567207	rs162556	rs10175368	rs163090	rs162330	rs162331
rs2551188								
rs2567206	0.8239							
rs2567207	0.6488	0.7494						
rs162556	0.2756	0.2536	0.2505					
rs10175368	0.7407	0.8216	0.6603	0.2308				
rs163090	0.1328	0.129	0.0902	0.037	0.1026			
rs162330	0.1059	0.118	0.0873	0.0287	0.0906	0.8121		
rs162331	0.1145	0.1249	0.1019	0.0476	0.1145	0.8837	0.8689	

### 3.4 Haplotype frequencies of CYP1B1 polymorphic sites

Haplotype frequencies of rs2551188‐rs2567206‐rs2567207‐rs10175368 and rs163090‐rs162330‐rs162331 in patients with prostate cancer were calculated and results are shown in Table 4. The major haplotype was G‐C‐T‐G for rs2551188‐rs2567206‐rs2567207‐rs10175368, which was expressed in 70.7% of healthy individuals overall. Interestingly, G‐C‐T‐G represents major allele at the respective rs sites and was significantly reduced in prostate cancer when compared to other haplotypes combined (*P* = .028). For rs163090‐rs162330‐rs162331, 2 haplotypes were similarly expressed and predominant, being T‐T‐G (47.7%) and A‐G‐A (46.6%) in overall controls. These haplotypes involving rs163090, rs162330 and rs162331, however, did not show any significant differences between cases and controls.

**Table 4:** Influence of lifestyle choices on frequencies of major haplotypes of rs2551188‐rs2567206‐rs2567207‐rs1017536 (G‐C‐T‐G) and rs163090‐rs162330‐rs162331 (A‐G‐A and T‐T‐G) of CYP1B1 between healthy controls and prostate cancer patients. Values expressed as fraction within group

Category	Haplotype	Control	Cancer	*P*‐value^a^
Overall	G‐C‐T‐G	0.7072	0.6563	**.028**
	T‐T‐G	0.4773	0.4609	.51
	A‐G‐A	0.4663	0.4895	.35
Non‐smoker	G‐C‐T‐G	0.6920	0.6563	.35
	T‐T‐G	0.4716	0.4486	.57
	A‐G‐A	0.4780	0.4997	.60
Smoker	G‐C‐T‐G	0.7178	0.6567	**.036**
	T‐T‐G	0.4810	0.4673	.66
	A‐G‐A	0.4586	0.4844	.41
Non‐drinker	G‐C‐T‐G	0.7112	0.6818	.37
	T‐T‐G	0.4602	0.4797	.59
	A‐G‐A	0.4742	0.4768	.94
Drinker	G‐C‐T‐G	0.7016	0.6407	.066
	T‐T‐G	0.4996	0.4523	.18
	A‐G‐A	0.4539	0.4926	.27

a *P*‐value based on chi‐square test and represents haplotype vs others combined within lifestyle group. *P* < .05 are in bold.

### 3.5 CYP1B1 polymorphisms within clinical stage of prostate cancer patients

Prostate cancer samples were classified in terms of clinical stage. There were 82 samples of unknown status. No statistical differences were observed when classified in terms of stage ≤2 (N = 253) vs ≥3 (N = 65) for all CYP1B1 polymorphic sites (data not shown).

### 3.6 Influence of lifestyle factors on risks of CYP1B1 polymorphisms for prostate cancer

As lifestyle factors can affect risks of prostate cancer, interaction between choices and CYP1B1 polymorphisms were determined. Table 5 shows risks of prostate cancer for polymorphisms analysed separately for smokers and non‐smokers. Interestingly among non‐smokers, none of the polymorphic sites were associated with cancer, whereas in smokers, genotype and allele type at rs2567206 (trend *P* = .061 and *P* = .032, respectively) and rs10175368 (*P* = .002 and *P* = .002, respectively) were risks for prostate cancer. Adjusted OR (95% CI) compared to major genotype was 1.41 (0.98 to 2.02) for C/T+T/T at rs2567206 and 1.75 (1.22 to 2.52) for G/A+A/A at rs10175368. Smoker status, however, did not affect risks of polymorphisms for stages of cancer (data not shown).

**Table 5:** Distribution of CYP1B1 polymorphisms (dominant pattern and allele) in healthy controls and prostate cancer patients among non‐smokers (A) and current or former smokers (B)

SNP ID	Genotype	Control n (%)	Cancer n (%)	Adj OR^a^ (95% CI)	Wald's test	Allele	Control n (%)	Cancer n (%)	OR (95% CI)	Fisher's exact
A. Non‐smokers										
rs2551188	G/G	84 (52.2)	63 (46.0)	Reference 1.29 (0.81‐2.05)	0.288	G	238 (73.9)	187 (68.2)	Reference 1.32 (0.92‐1.88)	0.146
	G/A+A/A	77 (47.8)	74 (54.0)			A	84 (26.1)	87 (31.8)		
rs2567206	C/C	87 (54.0)	62 (45.3)	Reference 1.45 (0.91‐2.33)	0.118	C	240 (74.5)	187 (68.2)	Reference 1.36 (0.95‐1.95)	0.101
	C/T+T/T	74 (46.0)	75 (54.7)			T	82 (25.5)	87 (31.8)		
rs2567207	T/T	81 (50.3)	67 (48.9)	Reference 1.10 (0.69‐1.75)	0.699	T	233 (72.4)	192 (70.1)	Reference 1.12 (0.78‐1.60)	0.586
	T/C+C/C	80 (49.7)	70 (51.1)			C	89 (27.6)	82 (29.9)		
rs162556	T/T	42 (26.1)	39 (28.5)	Reference 0.95 (0.57‐1.60)	0.854	T	167 (51.9)	147 (53.6)	Reference 0.93 (0.67‐1.29)	0.681
	T/C+C/C	119 (73.9)	98 (71.5)			C	155 (48.1)	127 (46.4)		
rs10175368	G/G	89 (55.3)	68 (49.6)	Reference 1.25 (0.78‐1.99)	0.352	G	245 (76.1)	192 (70.1)	Reference 1.36 (0.65‐1.95)	0.114
	G/A+A/A	72 (44.7)	69 (50.4)			A	77 (23.9)	82 (29.9)		
rs163090	T/T	39 (24.2)	33 (24.1)	Reference 1.02 (0.59‐1.75)	0.949	T	163 (50.6)	128 (46.7)	Reference 1.17 (0.85‐1.61)	0.366
	T/A+A/A	122 (75.8)	104 (75.9)			A	159 (49.4)	146 (53.3)		
rs162330	T/T	38 (23.6)	34 (24.8)	Reference 0.94 (0.55‐1.62)	0.829	T	160 (49.7)	132 (48.2)	Reference 1.06 (0.77‐1.47)	0.743
	T/G+G/G	123 (76.4)	103 (75.2)			G	162 (50.3)	142 (51.8)		
rs162331	A/A	38 (23.6)	40 (29.2)	Reference 0.72 (0.42‐1.22)	0.223	A	164 (50.9)	145 (52.9)	Reference 0.92 (0.66‐1.27)	0.681
	A/G+G/G	123 (76.4)	97 (70.8)			G	158 (49.1)	129 (47.1)		
B. Current or Former Smokers										
rs2551188	G/G	142 (58.2)	138 (52.5)	Reference 1.24 (0.87‐1.78)	0.236	G	374 (76.6)	381 (72.4)	Reference 1.25 (0.94‐1.66)	0.131
	G/A+A/A	102 (41.8)	125 (47.5)			A	114 (23.4)	145 (27.6)		
rs2567206	C/C	143 (58.6)	130 (49.4)	Reference 1.41 (0.98‐2.02)	0.061	C	375 (76.8)	373 (70.9)	Reference 1.36 (1.03‐1.80)	**0.032**
	C/T+T/T	101 (41.4)	133 (50.6)			T	113 (23.2)	153 (29.1)		
rs2567207	T/T	133 (54.5)	136 (51.7)	Reference 1.12 (0.78‐1.60)	0.549	T	368 (75.4)	374 (71.1)	Reference 1.25 (0.94‐1.65)	0.136
	T/C+C/C	111 (45.5)	127 (48.3)			C	120 (24.6)	152 (28.9)		
rs162556	T/T	56 (23.0)	70 (26.6)	Reference 0.76 (0.50‐1.15)	0.191	T	235 (48.2)	266 (50.6)	Reference 0.91 (0.71‐1.16)	0.451
	T/C+C/C	188 (77.0)	193 (73.4)			C	253 (51.8)	260 (49.4)		
rs10175368	G/G	153 (62.7)	127 (48.3)	Reference 1.75 (1.22‐2.52)	**0.002**	G	385 (78.9)	369 (70.2)	Reference 1.59 (1.20‐2.12)	**0.002**
	G/A+A/A	91 (37.3)	136 (51.7)			A	103 (21.1)	157 (59.7)		
rs163090	T/T	61 (25.0)	73 (27.8)	Reference 0.85 (0.57‐1.27)	0.418	T	252 (51.6)	261 (49.6)	Reference 1.08 (0.85‐1.39)	0.530
	T/A+A/A	183 (75.0)	190 (72.2)			A	236 (48.4)	265 (50.4)		
rs162330	T/T	63 (25.8)	66 (25.1)	Reference 1.01 (0.67‐1.52)	0.955	T	250 (51.2)	257 (48.9)	Reference 1.10 (0.86‐1.41)	0.452
	T/G+G/G	181 (74.2)	197 (74.9)			G	238 (48.8)	269 (51.1)		
rs162331	A/A	54 (22.1)	73 (27.8)	Reference 0.76 (0.50‐1.16)	0.203	A	239 (49.0)	266 (50.6)	Reference 0.94 (0.73‐1.20)	0.616
	A/G+G/G	190 (77.9)	190 (72.2)			G	249 (51.0)	260 (49.4)		

a OR adjusted for age, BMI, and drinker status. *P* < .05 are in bold.

Alcohol drinker status and its interaction with CYP1B1 polymorphisms and cancer risks are shown in Table 6. In non‐drinkers, none of the polymorphic sites were associated for prostate cancer with the exception for a trend for rs10175368 allele (*P* = .051). In contrast among drinkers, significant associations for cancer was observed for both genotypic and allelic frequencies at rs2567206 (*P* = .026 and *P* = .044, respectively) and rs10175368 (*P* = .012 and *P* = .016, respectively). At rs2567206, adjusted OR (95% CI) compared to major genotype was 1.57 (1.05 to 2.33) for C/T+T/T and at rs10175368, adjusted OR was 1.66 for G/A+A/A with a 95% CI of 1.12 to 2.47 compared to G/G. No effect on stage of cancer was observed, however, for the interaction between alcohol and CYP1B1 polymorphism.

**Table 6:** Distribution of CYP1B1 polymorphisms (dominant pattern and allele) in healthy controls and prostate cancer patients among non‐drinkers (A) and drinkers (B)

SNP ID	Genotype	Control n (%)	Cancer n (%)	Adj OR^a^ (95% CI)	Wald's test	Allele	Control n (%)	Cancer n (%)	OR (95% CI)	Fisher's exact
A. Non‐drinkers										
rs2551188	G/G	123 (56.7)	93 (53.1)	Reference 1.15 (0.77‐1.73)	0.494	G	333 (76.7)	256 (73.1)	Reference 1.21 (0.88‐1.67)	0.280
	G/A+A/A	94 (43.3)	82 (46.9)			A	101 (23.3)	94 (26.9)		
rs2567206	C/C	125 (57.6)	92 (52.6)	Reference 1.22 (0.81‐1.83)	0.339	C	334 (77.0)	255 (72.9)	Reference 1.24 (0.90‐1.72)	0.213
	C/T+T/T	92 (42.4)	83 (47.4)			T	100 (23.0)	95 (27.1)		
rs2567207	T/T	114 (52.5)	92 (52.6)	Reference 1.02 (0.68‐1.52)	0.942	T	323 (74.4)	253 (72.3)	Reference 1.12 (0.81‐1.53)	0.516
	T/C+C/C	103 (47.5)	83 (47.4)			C	111 (25.6)	97 (27.7)		
rs162556	T/T	48 (22.1)	43 (24.6)	Reference 0.85 (0.53‐1.37)	0.515	T	213 (49.1)	173 (49.4)	Reference 0.99 (0.74‐1.31)	0.943
	T/C+C/C	169 (77.9)	132 (75.4)			C	221 (50.9)	177 (50.6)		
rs10175368	G/G	133 (61.3)	93 (53.1)	Reference 1.40 (0.93‐2.11)	0.107	G	344 (79.3)	256 (73.1)	Reference 1.40 (1.01‐1.95)	0.051
	G/A+A/A	84 (38.7)	82 (46.9)			A	90 (20.7)	94 (26.9)		
rs163090	T/T	49 (22.6)	52 (29.7)	Reference 0.68 (0.43‐1.08)	0.104	T	218 (50.2)	177 (50.6)	Reference 0.99 (0.75‐1.31)	0.943
	T/A+A/A	168 (77.4)	123 (70.3)			A	216 (49.8)	173 (49.4)		
rs162330	T/T	47 (21.7)	48 (27.4)	Reference 0.69 (0.43‐1.11)	0.127	T	214 (49.3)	175 (50.0)	Reference 0.97 (0.73‐1.29)	0.886
	T/G+G/G	170 (78.3)	127 (72.6)			G	220 (50.7)	175 (50.0)		
rs162331	A/A	50 (23.0)	50 (28.6)	Reference 0.78 (0.49‐1.25)	0.305	A	224 (51.6)	175 (50.0)	Reference 1.07 (0.80‐1.41)	0.667
	A/G+G/G	167 (77.0)	125 (71.4)			G	210 (48.4)	175 (50.0)		
B. Drinkers										
rs2551188	G/G	101 (54.6)	108 (48.6)	Reference 1.32 (0.89‐1.96)	0.172	G	274 (74.1)	310 (69.8)	Reference 1.23 (0.91‐1.68)	0.185
	G/A+A/A	84 (45.4)	114 (51.4)			A	96 (25.9)	134 (30.2)		
rs2567206	C/C	103 (55.7)	100 (45.0)	Reference 1.57 (1.05‐2.33)	**0.026**	C	276 (74.6)	302 (68.0)	Reference 1.38 (1.02‐1.88)	**0.044**
	C/T+T/T	82 (44.3)	122 (55.0)			T	94 (25.4)	142 (32.0)		
rs2567207	T/T	98 (53.0)	111 (50.0)	Reference 1.18 (0.79‐1.74)	0.423	T	273 (73.8)	311 (70.0)	Reference 1.20 (0.89‐1.64)	0.242
	T/C+C/C	87 (47.0)	111 (50.0)			C	97 (26.2)	133 (30.0)		
rs162556	T/T	50 (27.0)	65 (29.3)	Reference 0.84 (0.54‐1.31)	0.438	T	188 (50.8)	236 (53.2)	Reference 0.91 (0.69‐1.20)	0.526
	T/C+C/C	135 (73.0)	157 (70.7)			C	182 (49.2)	208 (46.8)		
rs10175368	G/G	107 (57.8)	102 (45.9)	Reference 1.66 (1.12‐2.47)	**0.012**	G	281 (75.9)	303 (68.2)	Reference 1.45 (1.07‐1.98)	**0.016**
	G/A+A/A	78 (42.2)	120 (54.1)			A	89 (24.1)	141 (31.8)		
rs163090	T/T	51 (27.6)	54 (24.3)	Reference 1.19 (0.76‐1.86)	0.44	T	195 (52.7)	212 (47.7)	Reference 1.22 (0.93‐1.61)	0.181
	T/A+A/A	134 (72.4)	168 (75.7)			A	175 (47.3)	232 (52.3)		
rs162330	T/T	54 (29.2)	52 (23.4)	Reference 1.37 (0.87‐2.13)	0.171	T	194 (52.4)	214 (48.2)	Reference 1.19 (0.90‐1.56)	0.232
	T/G+G/G	131 (70.8)	170 (76.6)			G	176 (47.6)	230 (51.8)		
rs162331	A/A	41 (22.2)	60 (27.0)	Reference 0.75 (0.47‐1.18)	0.211	A	175 (47.3)	230 (51.8)	Reference 0.84 (0.63‐1.10)	0.206
	A/G+G/G	144 (77.8)	162 (73.0)			G	195 (52.7)	214 (48.2)		

a OR adjusted for age, BMI, and smoker status. *P* < .05 are in bold.

Interaction between lifestyle choices and CYP1B1 haplotypes was also determined. The effect of smoker and drinker status on prostate cancer risks for haplotype frequencies of rs2551188‐rs2567206‐rs2567207‐rs10175368 and rs163090‐rs162330‐rs162331 is shown in Table 4. Interestingly compared to healthy controls, major G‐C‐T‐G of rs2551188‐rs2567206‐rs2567207‐rs10175368 was significantly lower in cancer among smokers (*P* = .036) with a tendency for drinkers (*P* = .066), whereas no associations were observed in non‐smokers and non‐drinkers. Lifestyle factors did not influence the risks for any of the rs163090‐rs162330‐rs162331 haplotypes.

### 3.7 CYP1B1 polymorphisms and protein expression among BPH specimens

**Figure 1:**
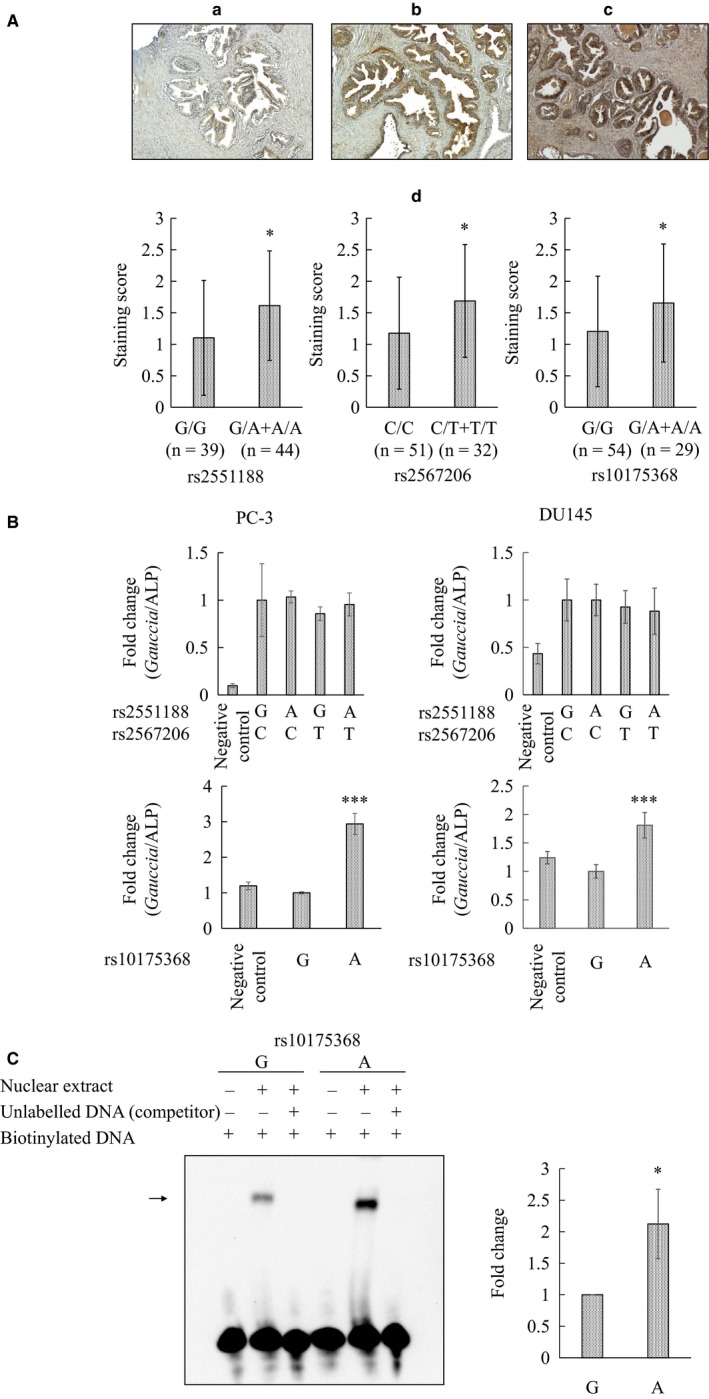
Properties of CYP1B1 promoter polymorphisms. A, CYP1B1 expression in clinical samples. Representative immunostaining of CYP1B1 by polymorphism status in benign prostatic hyperplasia (BPH) specimens. (a) None to weak staining, (b) moderate staining, and (c) stronger staining. (d) Staining score categorized by polymorphism status for rs2551188, rs2567206 and rs10175368. Score was significantly higher for minor genotype at rs2551188 (G/A+A/A), rs2567206 (C/T+T/T) and rs10175368 (G/A+A/A) compared to respective major genotypes. Number of specimens for each polymorphic status is indicated in parentheses. Data expressed as mean ± SD. *P < .05, determined by two‐tailed Mann‐Whitney U test. B, Effect of CYP1B1 polymorphisms on luciferase activity in prostate cancer cells. PC‐3 and DU145 cells seeded in 96‐well plates were transfected with either CYP1B1 minor or major allele constructs, and secreted *Gauccia* and ALP luciferase activities were measured. CYP1B1 promoter activities were calculated as the ratio of *Gauccia* to ALP. Levels were significantly higher for the rs10175368 allele A compared to major type G. Data expressed as mean±SEM for triplicates. ***P < .001, determined by Dunnett *t* (for rs2551188 and rs2567206) and two‐tailed Student *t* (for rs10175368) tests. C, Binding of nuclear factor to rs10175368 motif. Left: Nuclear extract prepared from DU145 cells was incubated with biotinylated double‐strand oligonucleotide probe for rs10175368 (−5346 to −5317 bp) with or without unlabelled DNA competitor, and complex separated by gel electrophoresis. Arrow denotes protein‐bound probe. Right: Stronger binding of protein factor was observed for the minor allele A compared to major allele G. Data expressed as mean±SEM for triplicates. *P = .045, two‐tailed Student *t* test

As genotypes and haplotypes were observed to be a risk for prostate cancer, expression level of CYP1B1 protein was evaluated for all sites. Immunohistochemistry was performed on 83 BPH specimens and scored. Interestingly compared to major genotype, variants at rs2551188 (*P* = .015), rs2567206 (*P* =.016) and rs10175368 (*P* = .047) were determined to have increased CYP1B1 levels (Figure 1A). Other polymorphic sites, however, showed no correlations with CYP1B1 expression (data not shown).

### 3.8 CYP1B1 polymorphisms and promoter activity

To assess functional properties, all polymorphic sites were evaluated for promoter activity by site‐directed mutagenesis followed by luciferase activity. Although minor alleles of rs2551188 and rs2567206 had no effect, rs10175368. A significantly up‐regulated CYP1B1 promoter activity compared to major allele G in both PC3 and DU145 cells (*P* < .001, Figure 1B). Other polymorphisms did not affect promoter activity (data not shown).

### 3.9 Nuclear extract binding of polymorphic site

Promoter activity was enhanced due to polymorphism, and thus, the binding capability of nuclear extracts to polymorphic sites was analysed. Nuclear extract was observed to weakly bind to rs10175368 G allele motif but interestingly, a 2.1‐fold larger amount of protein bound to the minor A allele form (*P* = .045, Figure 1C). Binding of nuclear extracts to motifs of rs2551188 and rs2567206 was not observed (data not shown).

## 4 DISCUSSION

The present study evaluated the risks of CYP1B1 polymorphisms in the promoter region/5′UTR for prostate cancer and the influence of lifestyle factors in a Caucasian population. Overall, drinking produced a risk for prostate cancer. This is in concordance with prior studies that show alcohol consumption to be associated with various forms of cancer^4^, ^10^ including prostate,^11^ and one of the main carcinogenic factors is acetaldehyde, which is derived from ethanol through cellular enzymes and CYP1B1 has been shown capable of this conversion.^21^ Studies demonstrate acetaldehyde to affect cellular properties such as DNA synthesis and repair,^24^ cause DNA mutations and adducts^24^, ^25^ and undergo further metabolism to form reactive radicals that can bind DNA.^26^ For smokers, although only a near trend was observed as a risk for prostate cancer in this study (*P* = .109), meta‐analysis shows a significant correlation for current smoking.^6^ A possible reason for the meagre significance may be due to the lack of information regarding the amount of smoking per individual and perhaps a breakdown by pack‐years may show a significant risk among heavy smokers, as a dose‐response effect of smoking was observed for prostate cancer mortality.^6^ Tobacco smoke contains numerous compounds that can promote cancer and agents such as polycyclic aromatic hydrocarbons (PAH) have been shown to enhance cancer of the lung, bladder, and head and neck.^12^ Interestingly in prostate cancer tissue, Caucasian ever smokers had significantly higher PAH‐DNA adducts compared to non‐smokers.^32^

When evaluating polymorphic sites of CYP1B1, the minor allele at rs2551188, rs2567206 and rs10175368 were an overall risk for prostate cancer. Interestingly when separated by lifestyle choices, none of these were associated with cancer among non‐smokers and non‐drinkers with the exception for a trend for rs10175368 allele among non‐drinkers. These results for men who do not smoke and drink alcohol are comparable to a study performed on Hispanic and non‐Hispanic Caucasians as no association for prostate cancer was observed for rs2551188 and rs2567206 by Beuten et al,^31^ although breakdown by lifestyle choices was not provided. On the contrary, smoking and alcohol drinking resulted in the minor alleles and genotypes at rs2567206 and rs10175368 to be a risk for cancer. Lifestyle choices can therefore modify the risks of CYP1B1 polymorphisms for prostate cancer.

Interaction between smoking and CYP1B1 polymorphisms leading to cancer has been shown in other studies. Harth et al^33^ analysed polymorphisms of genes in head and neck cancer and in a subgroup of non‐smokers, variants of the ERCC2 gene were predominant but when stratifying for smokers, CYP1B1 variant produced main effect. In lung adenocarcinoma, Rotunno et al^34^ observed no association for combined rs10175368/rs9341266 variants in never smokers but among ever smokers, this dual site was modified to significance. Timofeeva et al^35^ found that in women, no association was found for the minor genotype between rs1056836, rs1056827 and rs2567206 and lung cancer risk among non‐ and light smokers, whereas in heavy smokers, increased risk was observed. In a case‐only study of patients with colorectal cancer, Fan et al^36^ observed that risks of rs1056836 variant for cancer were increased in smokers compared to non‐smokers.

It is of interest that in a lifestyle choice study analysing 40 candidate genes among middle‐aged men, a CYP1B1 variant was associated with habitual alcohol drinking.^37^ Certainly, alcohol can also modify risks of variants for cancer as demonstrated by others. In cancer of the pharynx and larynx, although rs1056836 was a risk in non‐drinkers, odds ratio for the variant was much higher among drinkers.^38^ For squamous cell carcinoma of the head and neck, no association was found for polymorphisms of various genes but when analysed by potential modifiers, alcohol drinking affected the risks of CYP1B1 variants.^39^ In cervical cancer, rs1056836 variant was not a risk among non‐drinkers but among drinkers, a trend (*P* = .1) towards risk was observed with an OR value 2‐fold greater.^40^ Likewise in head and neck cancer, risk of rs10012 and rs1056837 had a crude OR of 1.6 and 2.3 for heterozygous and homozygous variant, respectively, but among alcohol drinkers, OR dramatically increased to 6.1 and 5.2, respectively.^41^

Linkage was observed among the 3 sites showing a risk for cancer along with rs2567207. Haplotype analyses demonstrate the major G‐C‐T‐G form to be significantly lower in prostate cancer cases compared to healthy controls. As was the case for individual polymorphic sites, lifestyle factors appear to interact with haplotype as G‐C‐T‐G proved to also have reduced risk among smokers and drinkers but not among abstainers of these choices. Haplotype of these sites may thus be an indicator of risk in the aetiology of prostate cancer that is influenced by lifestyle factors. Beuten et al^31^ also observed linkage between rs2551188 and rs2567206, and haplotype involving G‐C at these respective sites demonstrated a reduced risk for prostate cancer, which is in agreement with our results.

Polymorphisms of rs2551188, rs2567206 and rs10175368 are thus determined to be a risk for prostate cancer and the mechanism by which they may play a role is not known. These sites are located in the promoter or 5′UTR which are of importance as variants in this region may lead to increased gene expression^29^ and, consequently, increased enzyme or CYP1B1 levels. In concordance, results of this study demonstrate polymorphisms at these 3 sites to be associated with increased CYP1B1 protein levels as was observed in human prostatic specimens. On the contrary, only the rs10175368 minor allele showed increased luciferase activity and mobility shift demonstrated strong binding towards this variant. As these sites are linked, it may thus be through the minor allele of rs10175368 that expression levels of CYP1B1 are increased. This co‐dependence with rs10175368 may be pertinent for rs2567206 as cancer risk for these sites was modified by smoker and alcohol drinker status. This is corroborated in smokers as Rotunno et al^34^ observed rs10175368 to have increased mRNA expression among current smokers (*P*   =  .004) but not for never and former smokers. Thus, these lifestyle factors appear to interact at the genetic level to possibly increase CYP1B1 levels with rs10175368 playing a major role. Further experimentation is necessary to determine the identity of the factor that can bind to the rs10175368 minor allele in prostate cancer cells.

On the other hand, risk of the rs2551188 A allele was not affected by lifestyle factors and did not affect promoter activity or bind nuclear protein. Reasons for this independence are not known. Unlike rs2567206 and rs10175368 that are in the gene promoter region, rs2551188 is located in the 5′UTR of intron 1. This site undergoes a G to A base change and studies suggest this transition to affect RNA stability,^42^ which can consequentially lead to enhanced processing and gene or CYP1B1 expression. Alternatively, up‐regulation may be caused by linkage with rs10175368.

In conclusion, polymorphisms of the promoter and 5′UTR region of CYP1B1 are determined to be a risk for prostate cancer that can be modified by lifestyle factors in Caucasian men. These polymorphisms that potentially are capable of increasing gene expression levels and more so due to rs10175368 are thus critical to the prostate cell as CYP1B1 plays a role in the activation of carcinogens from precursors from various sources such as tobacco smoke and alcohol. These findings thus suggest CYP1B1 and its polymorphisms as a potential biomarker and gene of importance in understanding the pathogenesis of prostate cancer. It is essential to note, however, that this study does have its limitations as total sample size consisted of 405 controls and 400 prostate cancer cases, and a larger validation study is needed to verify results in the future.

## CONFLICT OF INTEREST

The authors confirm that there are no conflict of interests.

## AUTHOR CONTRIBUTIONS

TK, YH, RD and YT designed experiments. TK, YH, RW, YM, SM, IC, VS, SY, LT, SM and SS performed the experiments. TK, TD, RD and YT wrote the manuscript. All authors read and approved the final manuscript.
